# Measuring body satisfaction in women with eating disorders and healthy women: appearance-related and functional components in the Body Cathexis Scale (Dutch version)

**DOI:** 10.1007/s40519-021-01120-9

**Published:** 2021-02-16

**Authors:** Marlies E. Rekkers, Mia Scheffers, Jooske T. van Busschbach, Annemarie A. van Elburg

**Affiliations:** 1 Faculty of Social Sciences, Utrecht University, Heidelberglaan 1, 3584 CS Utrecht, The Netherlands; 2 School of Human Movement and Education, Windesheim University of Applied Sciences, Campus 2-6, 8017 CA Zwolle, The Netherlands; 3 Rob Giel Research Center, University Medical Center Groningen, University Center of Psychiatry, University of Groningen, P. O Box 30.001, CC72, 9700 RB Groningen, The Netherlands; 4 Centre for Eating Disorders, Altrecht Mental Health Institute, Rintveld, 3705WE Zeist, The Netherlands

**Keywords:** Body image, Body satisfaction, Body appreciation, Eating disorder, Body functionality

## Abstract

**Purpose:**

Differentiating the concept of body satisfaction, especially the functional component, is important in clinical and research context. The aim of the present study is to contribute to further refinement of the concept by evaluating the psychometric properties of the Dutch version of the Body Cathexis Scale (BCS). Differences in body satisfaction between clinical and non-clinical respondents are also explored.

**Method:**

Exploratory factor analysis (EFA) and confirmatory factor analysis (CFA) were used to investigate whether functional body satisfaction can be distinguished as a separate factor, using data from 238 adult female patients from a clinical sample and 1060 women from two non-clinical samples in the Netherlands. Univariate tests were used to identify differences between non-clinical and clinical samples.

**Results:**

EFA identified functionality as one of three factors, which was confirmed by CFA. CFA showed the best fit for a three-factor model, where functionality, non-weight, and weight were identified as separate factors in both populations. Internal consistency was good and correlations between factors were low. Women in the non-clinical sample scored significantly higher on the BCS than women with eating disorders on all three subscales, with high effect sizes.

**Conclusions:**

The three factors of the BCS may be used as subscales, enabling researchers and practitioners to use one scale to measure different aspects of body satisfaction, including body functionality. Use of the BCS may help to achieve a more complete understanding of how people evaluate body satisfaction and contribute to further research on the effectiveness of interventions focussing on body functionality.

**Level of evidence:**

Cross-sectional descriptive study, Level V.

## Introduction

The extensive interest in body satisfaction in the field of eating disorders entails an increasing need to differentiate and refine the concept of body satisfaction. Body satisfaction can be defined as satisfaction with appearance and/or functions of the body [1, 2] and body dissatisfaction is found to be a serious risk factor for the development, persistence and relapse of eating disorders [3–5]. Therefore, there is a need to measure body satisfaction, both in general and in terms of distinct components, to compare pre and post treatment outcomes and contrast these with body satisfaction as expressed in other populations. While the main emphasis in the field of eating disorders has been on appearance-related body satisfaction, particularly weight-related body parts, until recently functional body satisfaction has received insufficient consideration. With this in mind, more insight into different components of body satisfaction is of importance.

The Body Cathexis Scale (BCS), was one of the first scales to assess body satisfaction [6]. It was developed by Secord and Jourard [7] who defined body-cathexis as the degree of satisfaction or dissatisfaction with the various parts or processes of the body. The BCS assesses not only satisfaction with various parts of the body (including non-weight related body parts, such as eyes and hair, and weight-related body parts, such as hips and legs) but also satisfaction with bodily functions, such as energy level and coordination. This appreciation of bodily functions has recently received greater consideration [8, 9]. In the past the BCS has proved to be a valid and reliable questionnaire in various international psychometric studies [1, 2, 10–14], resulting in a Dutch translation of the BCS [15] and a first psychometric evaluation of the Dutch version in a non-clinical student sample [16]. Nowadays the BCS is a widely used questionnaire in The Netherlands to assess body satisfaction in clinical practice, because the questionnaire is gender neutral and suitable to use in different mental disorders, where body dissatisfaction may play a role, such as, next to eating disorders, somatic symptom disorders, body dysmorphic disorder, anxiety disorders, trauma-related disorders, and mood disorders [17]. However, the Dutch version of the BCS still lacks state-of-the-art psychometric evaluation, in particular exploratory and confirmatory factor analyses, in both clinical and representative non-clinical samples.

Following the example of the BCS, other questionnaires were developed to measure body satisfaction, such as the Body Esteem Scale (BES) [18], the Body Shape Questionnaire (BSQ) [19] and the Body Dissatisfaction Scale (BDS) [20]. In 2002 Thompson and Berg [21] stated that body satisfaction needed additional refinement using different components of body satisfaction, such as weight satisfaction, shape satisfaction and satisfaction with specific body parts and features. In the field of eating disorders, this resulted in reduced attention to general body satisfaction and greater attention to satisfaction with weight and shape [22, 23].

In addition to differentiating various components of body satisfaction, over the past decade another development has been crucial in refining the concept of body satisfaction. Instead of a “pathology driven” approach, positive adaptive or healthy aspects of body satisfaction have received increasing emphasis [9, 24–26]. Frisén and Holmqvist [27] used a qualitative design to study positive body image attributes in Swedish adolescents and discovered that besides acceptance of the body, functional perception of the body is an important ingredient of body satisfaction. They concluded that encouraging mindsets evaluating the body more for function than appearance might help increase positive body satisfaction. Wood-Barcalow et al. [28] also identified a functional attitude towards one’s body as one of the attributes of a positive evaluation of the body. In the same line Halliwell [29] observed that functional aspects of body image may serve as a protective psychological mechanism against body dissatisfaction. In this context Wood-Barcalow et al. [28] stated that additional instruments measuring different positive attributes of body satisfaction are needed. Furthermore, Alleva et al. [9] emphasized the need for validated questionnaires measuring body functionality to drive and improve body satisfaction studies and introduced the seven-item unidimensional Functionality Appreciation Scale (FAS) [30]. However, it seems worthwhile to renew attention to the strength of the already available BCS, an instrument measuring both aesthetic and functional body satisfaction. For this reason, updated psychometric information, regarding the factor structure of the BCS may expand the value of the BCS in clinical practice, particularly with respect to eating disorders.

The first aim of the present study is to evaluate the psychometric properties of the Dutch version of the 40-item BCS [16], by re-examining the factor structure and investigate the hypothesis that functional body satisfaction is a distinguishable factor in all samples. The second aim is to explore differences in body (dis)satisfaction between clinical and non-clinical data. In line with earlier studies [2, 24, 31], it is hypothesized that the BCS will reveal a significantly lower body satisfaction, for the total scale and the subscales, in female patients with eating disorders compared to women in a non-clinical sample.

## Method

### Participants

Three independent samples were used in this study: one clinical sample and two non-clinical samples. The clinical sample consisted of 238 adult female patients with a variety of eating disorders. The patients were diagnosed according to DSM-IV criteria in the following categories: 86 (36.1%) with anorexia nervosa (AN); 52 (21.8%) with bulimia nervosa (BN) and 100 (42.0%) with eating disorder not otherwise specified (EDNOS). According to the DSM-5 criteria, 22 participants (9.2% of the total) diagnosed with EDNOS could have been diagnosed with binge eating disorder (BED). All patients attended an outpatient clinic specializing in the treatment of eating disorders in The Netherlands. The two non-clinical samples were recruited online and consisted of 579 (sample one) and 481 (sample two) adult women from the general Dutch population.

### Procedure

In the period from 2007 until 2019, patients in the clinical sample filled out the BCS as part of assessment before starting treatment. Data collection for the two non-clinical samples was conducted using a snowball sampling method through e-mails sent to potential participants in the network of students at the Department of Human Movement Sciences, Vrije Universiteit Amsterdam in 2016 (sample one) and the network of one student at the Master Youth Studies Utrecht University in 2019 (sample two). The e-mail included a link to the questionnaires, information about the study objective and the voluntary and anonymous participation, and a request to readers to forward the e-mail to others in their network. No participatory incentives were offered. Participants completed the questionnaire through a secure online system. All survey materials were removed from the internet upon completion of the data collection phase. This procedure was approved of the medical ethics review committee of the VU University Amsterdam (sample one) and the medical ethics review committee of the University Medical Centre Groningen (sample two). Informed consent was obtained from all individual participants included in this study, both clinical and non-clinical, authorizing anonymous use of their scores on the BCS for research purposes.

### Measure

The BCS [7] measures the degree of satisfaction with appearance and functionality of different parts of the body. The scale comprises 40 items, scored on a five-point Likert scale ranging from 1, “very dissatisfied”, to 5, “very satisfied”. Construct validity and concurrent validity of the English 40-item BCS are good [10, 12, 14]. The original English version was translated into Dutch and psychometrically evaluated by Baardman and De Jong [15]. Dorhout et al. [16] further evaluated the Dutch 40-item version and found good reliability (Cronbach’s alpha 0.91) and construct validity (Body Image Visual Analogue Scale: *r* = 0.68 (*p* < 0.01); Rosenberg Self Esteem Scale: *r* = 0.47 (*p* < 0.01)).

### Statistical analyses

Exploratory factor analysis (EFA) was performed with the BCS data from non-clinical sample one and the clinical sample. Maximum likelihood with oblique rotation was used as the factor extraction method [32] according to SPSS 20.0. Numbers of factors retained were based on interpretation of the scree plot [33] and parallel analysis [34]. Interpretability of the factors [35] and theoretical considerations [36] were used to redefine factor structures. Cross-loadings were defined as an item that loads at > 0.32 on two or more factors [35].

As is generally recommended [37–39] we used a second independent non-clinical sample for confirmatory analysis (CFA) using Mplus Version 8.0 [40] to evaluate the adequacy of the proposed factor structure following from EFA. Because each type of index provides different information about model fit [37], we chose to report a broad range of indices and included root mean square error of approximation (RMSEA), standardized root mean square residual (SRMR), Comparative Fit Index (CFI) and Tucker Lewis index (TLI). The RMSEA represents the fit of the estimated covariance matrix to the population’s covariance matrix [41]. The RMSEA is regarded as one of the most informative fit indices thanks to its sensitivity to the number of estimated parameters in the model, which enables it to favour parsimonious models. As a rule of thumb, RMSEA values < 0.08 suggest adequate and < 0.05 good model fit [42]. The SRMR is the standardized square root of the difference between the residuals of the sample covariance matrix and the hypothesised covariance model. An SRMR between 0.05 and 0.10 indicates an acceptable fit and values < 0.05 indicate good fit [43]. The CFI [44] compares the sample covariance matrix with a null model of uncorrelated latent variables. The CFI is one of the most commonly reported fit indices, as it is one of the measures least affected by sample size and is often reported together with the TLI, a comparative fit index slightly differing from the CFI in its approach to sample size and handling of the effect of model complexity [40]. CFI and TLI values in the range between 0.90 and 0.95 may be regarded as indicating good model fit [37].

Independent *t* tests were used to analyze differences in scores between the non-clinical and the clinical sample. Cohen’s *d* was used to establish effect sizes.

## Results

No significant differences in age and BMI were found between the clinical sample (*n* = 238) and non-clinical sample one (*n* = 579). Mean age was 26.23 (*SD* 7.16, range 18–62) in the clinical sample and 27.45 (*SD* 12.25, range 18–66) in non-clinical sample one, *t*(721,801) = 1.767, *p* = 0.078. Mean BMI was 21.91 (*SD* 4.82) and 22.15 (*SD* 2.88), respectively, *t*(307.482) = 0.698, *p* = 0.486.

### Factor analyses

The Kaiser–Meyer–Olkin (KMO) scale verified the sampling adequacy for the first EFA on non-clinical sample one, KMO = 0.917 (“good”, according to Field [45]); Barlett’s test of sphericity was statistically significant (*χ*^2^ = 8329; *df* = 780, *p* < 0.0001), indicating that data were suitable for EFA. Parallel analysis [46] and inspection of the scree plot were employed to determine the appropriate number of factors to retain. The parallel analysis showed factor solutions with eigenvalues ranging from 1237 to 1589 for the first ten factors. This confirms the decision to retain the first three factors that could be distinguished in our data with all eigenvalues above this maximum. Also, the scree plot leaves no room for misinterpretation. The scree plot showed an inflection justifying retaining three factors. This three-factor solution accounted for 34.39% of the variance.

In the EFA in the sample of women with eating disorders KMO was 0.859 (“good” according to Field [45]) and Barlett’s test of sphericity was statistically significant (*χ*^2^ = 3377; *df* = 780, *p* < 0.0001). The scree plot showed an inflection justifying retaining three factors, accounting for 31.85% of the variance.

In both EFA’s, the same three-factor solution offered the best fit (see Table 1). Factor 1 (20 items: 38, 15, 13, 6, 40, 33, 30, 24, 21, 5, 14, 19, 2, 22, 23, 1, 4, 18, 36, 29) consisted of non-weight related items; factor 2 (seven items: 17, 16, 39, 10, 26, 28, 3) comprised weight-related items, and factor 3 (13 items: 7, 11, 9, 35, 31, 34, 27, 37, 20, 25, 32, 12, 8) referred to functionality. Item 12 “back” had low loadings on all factors; in the clinical as well as in non-clinical sample one. We decided to list item 12 under the factor functionality, since it loaded highest on this factor in the larger non-clinical sample. Item 8 “elimination” also loaded low on all factors, but highest on the factor functionality. Item 22 “arms” is the only item showing different results on the loadings: in the non-clinical sample the loading was highest on the factor non-weight, whereas in the clinical sample the loading was highest on the factor weight. We decided to let the non-clinical findings be leading.

**Table 1:** Exploratory factor analyses: item loadings on the three factors in non-clinical sample one and the clinical sample

Sample		Non-clinical one (*n* = 579)			Clinical (*n* = 238)		
Factor		1	2	3	1	2	3
BCS 38	Face	**0.650**	− 0.192	0.073	**0.732**	− 0.086	0.003
BCS 15	Chin	**0.549**	− 0.103	0.043	**0.487**	− 0.245	− 0.001
BCS 13	Ears	**0.543**	0.062	− 0.011	**0.575**	0.139	− 0.057
BCS 6	Nose	**0.518**	0.058	0.077	**0.529**	0.056	− 0.073
BCS 40	Sex organs	**0.508**	− 0.148	− 0.047	**0.391**	0.086	− 0.107
BCS 33	Voice	**0.508**	− 0.089	− 0.039	**0.576**	− 0.017	0.021
BCS 30	Overall appearance	**0.507**	− 0.364	− 0.015	**0.465**	− 0.457	− 0.048
BCS 24	Eyes	**0.505**	0.057	− 0.072	**0.514**	− 0.038	0.002
BCS 21	Shoulder Width	**0.484**	− 0.077	− 0.046	**0.313**	− 0.238	− 0.032
BCS 5	Body hair	**0.484**	0.022	0.050	**0.322**	0.068	− 0.148
BCS 14	Age	**0.480**	0.046	0.038	**0.292**	− 0.015	− 0.199
BCS 19	Keenness of senses	**0.450**	0.180	− 0.095	**0.293**	− 070	− 0.250
BCS 2	Facial complexion	**0.416**	0.070	− 0.076	**0.500**	0.055	− 0.029
BCS 22	Arms	**0.412**	− 0.242	− 0.073	**0.210**	0.465	− 0.020
BCS 23	Breasts	**0.408**	− 0.126	− 0.082	**0.347**	− 0.106	0.061
BCS 1	Hair	**0.395**	− 0.040	− 0.028	**0.420**	− 0.053	0.056
BCS 4	Hands	**0.375**	− 0.063	0.022	**0.270**	− 0.036	0.003
BCS 18	Height	**0.361**	− 0.150	− 0.002	**0.323**	− 0.113	− 0.019
BCS 36	Knees	**0.211**	− 0.112	− 0.157	**0.315**	− 0.257	0.030
BCS 29	Teeth	**0.210**	− 0.078	0.001	**0.460**	− 0.076	− 0.050
BCS 17	Profile	0.093	− **0.861**	− 0.007	0.039	− **0.830**	− 0.042
BCS 16	Build	0.095	− **0.790**	− 0.012	0.066	− **0.759**	− 0.018
BCS 39	Weight	− 0.035	− **0.737**	− 0.134	− 0.091	− **0.729**	− 0.099
BCS 10	Waist	− 0.086	− **0.631**	− 0.224	0.100	− **0.500**	− 0.063
BCS 26	Hips	0.266	− **0.542**	− 0.019	0.215	− **0.612**	0.110
BCS 28	Legs	0.252	− **0.462**	− 0.079	0.149	− **0.546**	0.041
BCS 3	Appetite	0.183	− **0.388**	− 0.148	0.181	− **0.449**	− 0.159
BCS 7	Physical stamina	− 0.167	− 0.062	− **0.787**	− 0.117	− 0.207	− **0.669**
BCS 11	Energy level	− 0.106	− 0.039	− **0.738**	− 0.041	− 0.076	− **0.658**
BCS 9	Muscle strength	− 0.167	− 0.124	− **0.731**	0.005	− 0.065	− **0.654**
BCS 35	Physical skills	0.059	− 0.038	− **0.707**	0.015	0.011	− **0.719**
BCS 31	Muscle tone	− 0.007	− 0.221	− **0.579**	0.143	− 0.229	− **0.396**
BCS 34	Health	0.223	0.038	− **0.541**	0.027	0.081	− **0.547**
BCS 27	Resistance to illness	0.201	0.153	− **0.428**	− 0.056	− 0.113	− **0.401**
BCS 37	Flexibility	0.168	− 0.112	− **0.397**	0.140	− 0.021	− **0.452**
BCS 20	Pain tolerance	0.274	0.173	− **0.365**	0.028	0.119	− **0.503**
BCS 25	Coordination	0.359	0.059	− **0.322**	0.231	0.083	− **0.427**
BCS 32	Sleep	0.140	− 0.060	− **0.263**	0.253	0.117	− **0.252**
BCS 12	Back	0.234	− 0.040	− **0.293**	0.211	− 0.222	− **0.210**
BCS 8	Elimination	0.134	− 0.113	− **0.235**	0.100	− 0.117	− **0.293**

Boldface indicates highest factor loadings

CFA using non-clinical sample two provided the best fit for the three-factor model that resulted from the EFA (see Table 2). Fit could be improved by permitting correlated errors for items 31 “muscle tone” and 9 “muscle strength” (Modification Index 84.970) and for items 34 “health” and 27 “resistance to illness” (Modification Index 88.291).

**Table 2:** Confirmatory factor analysis of non-clinical sample two (*n* = 481)

Model		*χ*^2^	*df*	RMSEA (90% CI)	SRMR	CFI	TLI
1	1 factor	3174	740	0.083 (0.080–0.086)	0.076	0.625	0.604
2	3 factors	2033	737	0.060 (0.057–0.064)	0.064	0.800	0.788
3	3 factors:31 with 9; 34 with 27	1863	735	0.057 (0.053–0.060)	0.062	0.826	0.815

*χ*^2^ Chi square, *df* degrees of freedom, *RMSEA* root mean square error of approximation, *90% CI* 90% confidence interval of the RMSEA; *SRMR* standardized root mean square residual, *CFI* comparative fit index, *TLI* Tucker Lewis index

### Internal consistency and correlations

Cronbach’s alpha’s in non-clinical sample one and in the clinical sample were, respectively, 0.92 and 90 for the total scale, 0.84 and 0.85 for factor 1 (non-weight), 0.85 and 0.83 for factor 2 (functionality) and 0.86 and 0.83 for factor 3 (weight).

Correlations in non-clinical sample one between the different factors were 0.53 between non-weight and weight, 0.57 between non-weight and functionality, and 0.53 between weight and functionality. In the clinical sample correlations between the different factors were 0.49 between non-weight and weight, 0.55 between non-weight and functionality and 0.35 between weight and functionality.

### Differences between groups

Differences between the scores in the clinical sample and in non-clinical sample one were significant (*p* < 0.001) for BCS total mean score as well as for the three subscales, meaning that in non-clinical sample one, women showed more satisfaction with their body than women in the clinical sample. The effect sizes were high, with the subscale Weight showing the highest effect size (see Table 3).

**Table 3:** Means (*M*) and standard deviations (*SD*) of scores on the Body Cathexis Scale and factors in the clinical sample of females with eating disorders and of in non-clinical sample one, test of the difference and effect size (Cohen’s *d*)

(Sub) Scale	Eating Disorders (*n* = 238)	Non-clinical one (*n* = 579)	*t* (728)	Cohen’s *d*
	*M* (*SD*)	*M* (*SD*)		
BCS total mean score	2.88 (0.49)	3.58 (0.49)	18.75*	1.43
Non-weight	3.10 (0.53)	3.67 (0.49)	14.64*	1.12
Weight	2.03 (0.73)	3.41 (0.79)	23.03*	1.81
Functionality	3.01 (0.61)	3.56 (0.60)	11.72*	0.91

*p < 0.001

## Discussion

This study had two principal aims. The first aim was to investigate the hypothesis that functional body satisfaction is a distinctive factor in the BCS, in both non-clinical and clinical samples. EFA did indeed identify Functionality as one of the three factors, and this was confirmed by CFA. More specifically, the CFA results revealed adequate fit values for the three-factor model with a Functionality, a Non-weight and a Weight factor. These three factors may be used as subscales, given their good internal consistency and the relatively low correlations between the factors. The high alpha for the total scale is in concordance with earlier research [1, 11, 14, 16].

Interestingly, in the clinical sample the correlations between the factors are lower than in the non-clinical sample, especially between the factors functionality and weight. An explanation for these results might be that patients with eating disorders, due to their negative body image, focus to a high degree on a limited area of body satisfaction, while subjects with a more positive body image may be expected to have a broader and more integrated perception of body appreciation. In the same vein Tylka and Wood-Barcalow [47] concluded that a positive body satisfaction is not limited to one dimension of body appreciation. They regard positive body image as a holistic construct. It might be possible that patients with eating disorders have lost an integrated and holistic view of their body and that it is important to re-establish this view in therapy.

Having the option of using three distinct subscales may enhance research, assessment and treatment of different components of body satisfaction [28, 48, 49], in particular body functionality [8, 9, 50]. Abbott and Barber [8] observed that women do not automatically mention their body’s functionality when asked to reflect on or evaluate their bodies and they also found that when functionality is incorporated into the measurement of body image, the functionality of the body is valued more highly than appearance by both male and female adolescents. These findings highlight even more the importance of measuring the functional dimension of body satisfaction. Already in 2011 Cash and Smolak [51] mentioned the lack of research on body functionality. The present study provides evidence that the BCS fills this gap and does incorporate a body functionality subscale. Therefore, the BCS could be a valuable instrument for assessing functional body satisfaction and thus help to achieve a more complete and holistic understanding of how people evaluate their body. Given the fact that Alleva et al. [30] recently developed the FAS to specifically measure body functionality, it would be relevant to investigate in both clinical and non-clinical samples to what extent the subscale Functionality of the BCS and the FAS measure the same construct.

The second aim of this study was to explore differences in body satisfaction between the clinical and the non-clinical samples. As predicted, women in the non-clinical sample reported significantly greater satisfaction with their bodies than those in the clinical sample, as reflected by differences on total scores and subscale scores with high effect sizes. The subscale Weight showed the highest effect size (*d* = 1.81). This result is not surprising, since dissatisfaction and obsession with weight-related body characteristics and body parts is a key issue in patients with eating disorders [52]. The effect sizes for the subscales Functionality (*d* = 0.91) and Non-weight (*d* = 1.12), though still high, were lower than for the subscale Weight. Functional body satisfaction reflected the least relative difference. The results suggest that it might be worthwhile to investigate whether enhancing functional satisfaction, as suggested by Frisén and Holmqvist [27], could lead to a generally more positive body image in female patients with eating disorders. However, functional body image is often not discussed in treatment, because aesthetic body image is generally the main problem presented by patients. When professionals and patients become more conscious of body functionality, using questionnaires assessing body functionality like the BCS, this may also provide a basis for therapeutic interventions to mitigate body dissatisfaction by focusing on body functionality. Within this context, Webb et al. [49] state that recognizing and appreciating the various functions that the body performs can be a valuable resource for enhancing positive body image. In line with this statement, Alleva et al. [53] recently found that focusing on body functionality was effective in protecting and promoting a positive body image in female students.

The present study has several limitations. First of all, emphasis was put on factor-analytical approaches, because we wanted to investigate the hypothesis that functional body satisfaction is a distinguishable factor in the clinical and the non-clinical samples. Establishing test–retest reliability and construct validity, especially in clinical populations, needs to follow, now this hypothesis has been confirmed. We also could not evaluate whether the BCS items are invariant across the non-clinical and clinical groups, because the size of the clinical sample was too small for multiple group CFA. Another limitation concerns the composition of the samples used. Since the average age of the female participants in all samples was mid-twenties, it limits the generalizability of these findings to other samples. Additional research with male and older participants is desirable.

## Conclusion

The BCS has long been used as a general measure for body satisfaction. Other measurements were developed after the BCS with a focus predominantly on weight and shape. In the past decade, awareness has increased that other components of body satisfaction, such as functional body satisfaction, should be measured as well. This led to new scales to assess body functionality but also to a renewed interest in the BCS which was assumed to be a reliable and valid instrument that also incorporates body functionality. In this study of the Dutch version of the BCS this was confirmed with three factors identified: non-weight, weight, and functionality. These three factors may be used as subscales, enabling mental health professionals and researchers to use one scale to measure different aspects of body satisfaction, including body functionality. These results may stimulate new perspectives on body image therapy and enhance our understanding of how body satisfaction in female patients with eating disorders differs from healthy women.

## What is already known on this subject?

The BCS measures both aesthetic and functional body satisfaction. However, despite relevant psychometric studies in the past the BCS lacks state-of-the-art psychometric evaluation.

## What this study adds?

Factor analyses revealed a three-factor model (functionality, non-weight and weight). Using these factors as subscales may enhance assessment and treatment of different components of body satisfaction.
